# Aristolochic acid induces acute kidney injury through ferroptosis

**DOI:** 10.3389/fphar.2024.1330376

**Published:** 2024-03-27

**Authors:** Xuan Huang, Ruihua Liu, Cuixia Zhan, Haishan Wu, Jinjin Fan, Zhijian Li, Xiao Yang

**Affiliations:** ^1^ Department of Nephrology, The First Affiliated Hospital, Sun Yat-sen University, Guangzhou, China; ^2^ Key Laboratory of Nephrology, National Health Commission and Guangdong Province, Guangzhou, China

**Keywords:** acute kidney injury, aristolochic acid, ferroptosis, GPx4, lipid peroxidation

## Abstract

Aristolochic acid (AA)-induced acute kidney injury (AKI) presents with progressive decline in renal function and rapid progression to end-stage renal disease. Among the multiple mechanisms identified in AKI, ferroptosis has been shown to be involved in various forms of AKI. But few studies have elucidated the role of ferroptosis in AA-induced AKI. In this study, we investigated the role of ferroptosis in AA-induced acute renal tubular injury *in vivo* and *in vitro*. Mice with acute aristolochic acid nephropathy showed increased malondialdehyde levels, aggravated lipid peroxidation, decreased superoxide dismutase activity, and glutathione depletion. The expression of glutathione peroxidase 4 was decreased and the expression of acyl-CoA synthetase long-chain family member 4 was increased. Inhibition of ferroptosis by ferrostatin-1 significantly improved the renal function, reduced histopathological lesions, partially alleviated lipid peroxidation, and restored the antioxidant capacity. *In vitro* studies also revealed that AA significantly reduced cell viability, induced reactive oxygen species production, increased intracellular iron level and decreased ferroptosis-related protein expression. Inhibition of ferroptosis significantly increased cell viability and attenuated AA-induced renal tubular epithelial cell injury. It is suggested that ferroptosis plays an important role in AA-induced acute tubular injury. And inhibition of ferroptosis may exert renoprotective effects possibly by preventing lipid peroxidation, restoring the antioxidant activity or regulating iron metabolism.

## 1 Introduction

Acute kidney injury (AKI) is a common critical disease characterized by rapid decline of renal function in a short period of time. Renal ischemia and nephrotoxic agents are the most common causes of AKI. The prevalence of AKI accounts for 10%–15% of all hospitalizations and can exceed 50% in the intensive care unit (Hoste et al., 2015; Al-Jaghbeer et al., 2018). The mortality of AKI in hospitalized patients who do not recover is as high as 50% (Siew et al., 2016), and the risk of AKI progressing to chronic kidney disease (CKD) or even end-stage renal disease (ESRD) is significantly higher (See et al., 2019). Aristolochic acid nephropathy (AAN) is a tubulointerstitial disease caused by the ingestion of herbal medicines containing aristolochic acid (AA). New cases of AAN continue to be reported despite strict bans against AA in countries around the world (Anandagoda and Lord, 2015), and no effective treatment is available for AAN till now (Jadot et al., 2017). Acute kidney injury accounts for 4.3%–22% of the clinical types of AAN, manifesting as interstitial nephritis, inflammatory infiltrates and acute tubular necrosis, with progressive decline in renal function and rapid progression to ESRD in most patients (Chen et al., 2012; Yang et al., 2012).

Multiple cell death pathways are involved in the AKI pathogenesis, including apoptosis, necrosis, and the recently proposed regulated necrosis (Linkermann and Green, 2014), but the exact molecular mechanisms are yet unknown. In AKI, necrotic renal tubular cells are usually the source of damage-associated molecules patterns (DAMPs) (Kaczmarek et al., 2013), thus regulated necrosis is thought to play a key role in AKI. Ferroptosis is a newly described non-apoptotic form of regulated cell death, which is caused by iron-dependent lipid peroxidation (Dixon et al., 2012). It dependents on iron and reactive oxygen species, and has been shown to be associated with a variety of pathological states and diseases, such as neurodegenerative diseases, ischemia-reperfusion injury, cancer, infections, and immune diseases (Skouta et al., 2014). Deficiency of the key endogenous inhibitor of ferroptosis, glutathione peroxidase 4 (GPX4), would lead to AKI in mice, while ferrostatin-1 (Fer-1), an inhibitor of ferroptosis, attenuates renal injury by scavenging lipid peroxidation *in vivo* (Friedmann et al., 2014). In addition, an important role of ferroptosis has been demonstrated in rhabdomyolysis, ischemic, nephrotoxic-induced AKI, and other AKI models. However, the role of ferroptosis in AA-induced AKI has not been fully studied. In this study, we intended to investigate the role of ferroptosis and its possible mechanisms in AA-induced acute renal tubular injury by *in vivo* and *in vitro* experiments.

## 2 Materials and methods

### 2.1 Reagents and antibodies

AA Ⅰ and Fer-1 was purchased from Sigma-Aldrich (St. Louis, MO) and prepared as a stock solution in dimethyl sulfoxide (Sigma-Aldrich). Rabbit anti-GPX4 antibody and anti-ACSL4 antibody was purchased from Abcam (Cambridge, MA). Mouse anti-β-actin antibody, horseradish peroxidase (HRP)-conjugated anti-mouse IgG and HRP-conjugated anti-rabbit IgG were purchased from Cell Signaling Technology (Beverly, MA).

### 2.2 Animals

All procedures were conducted in accordance with the protocol approval by the Ethics Committee of the First Affiliated Hospital, Sun Yat-sen University (Guangzhou, China). SPF-grade 8-week-old male C57BL/6J mice were obtained from Gempharmatech Co., Ltd (Jiangsu, China). Mice were randomly divided into control group, AA group and AA + Fer-1 group. 5 mg/kg AA was injected intraperitoneally for five consecutive days, and 5 mg/kg Fer-1 or vehicle was injected 30 min before AA injection. All mice were euthanized 12 h after the last injection, and blood samples and kidney samples were collected.

### 2.3 Renal histopathology

The kidney tissues were fixed in 4% paraformaldehyde at 4°C overnight, washed with PBS, dehydrated in graded ethanol, embedded in paraffin, and cut into sections for hematoxylin and eosin (HE) staining and periodic acid-Schiff (PAS) staining. Histological changes were quantitated by the percentage of tubules that showed cell necrosis, loss of brush border, cast formation, and tubule dilatation as acute tubular necrosis (ATN) score: 0, none; 1, <10%; 2, 11%–25%; 3, 26%–45%; 4, 46%–75%; and 5, >76% (He et al., 2008). At least 5–10 fields (×200) were reviewed for each slide. For transmission electron microscopy, the kidney tissues were fixed in 2% osmium-glutaraldehyde at 4°C for further fixation, dehydration, embedding, sectioning and staining. For quantitative analysis of mitochondria, the mitochondrial length was measured in an area of approximately 100 μm^2^ in each group using Image J.

### 2.4 Assessment of lipid peroxidation

The malondialdehyde (MDA) levels were detected using the MDA assay kit (Nanjing Jiancheng Bioengineering Institute, Nanjing, China) with TBA method, and the superoxide dismutase (SOD) activity was measured using the SOD assay kit (Nanjing Jiancheng Bioengineering Institute) with WST-1 method. The glutathione (GSH) levels were assessed with the GSH assay kit (Nanjing Jiancheng Bioengineering Institute) according to the manufacturer’s instructions.

### 2.5 Western blotting analysis

Kidney and cellular proteins were extracted with lysis buffer (Cell Signaling Technology, Beverly, MA) containing a protease/phosphatase inhibitor cocktail. The protein concentration was determined using a BCA protein assay kit (Thermo Scientific, Fresno, CA). Prepared protein samples were then loaded and separated by 10%–15% SDS-PAGE gel. After electrophoresis, samples were electrotransferred to PVDF membranes (EMD Millipore, Billerica, MA). Membranes were blocked with 5% (w/v) nonfat dry milk in tris buffered saline with tween 20 (TBST) for 1 h, and incubated with rabbit anti-GPX4 antibody (1:1000), anti-ACSL4 antibody (1:1000), or mouse anti-β-actin antibody (1:5000), respectively, diluted in TBST with 5% bovine serum albumin (BSA) overnight at 4°C, followed by incubation with the species-appropriate horseradish peroxidase (HRP)-conjugated secondary antibody (1:5000). Blots were detected using chemiluminescence imaging system (Clinx Science Instrument, Shanghai, China). Densitometric analysis was performed using ImageJ and normalized by β-actin.

### 2.6 Immunofluorescence analysis

The paraffin-embedded kidney sections were dewaxed and rehydrated. After antigen retrieval, the sections were incubated with block buffer (5% BSA in PBS) for 1 h at room temperature. The sections were incubated with anti-GPX4 (1:25), anti-ACSL4 (1:25) antibody and anti-TOM20 (1:400) antibody, respectively, at 4°C overnight, followed by Alexa Fluor 488-conjugated anti-goat IgG (1:1000) antibody. Tissues were counterstained with the fluorescent dye DAPI (1:200) for 5 min. All images were taken using a laser scanning confocal microscope (Zeiss LSM 710, Carl Zeiss, Germany).

### 2.7 Cell culture

NRK-52E cells were obtained from American Type Culture Collection (ATCC, Manassas, VA) and maintained in Dulbecco’s modified Eagle medium: nutrient mixture F-12 (DMEM/F12) (Gibco, Fresno, CA) supplemented with 5% fetal bovine serum (FBS) and 100 U/mL penicillin-streptomycin solution. Cells were cultured in a 37°C humidified incubator with 5% CO_2_. To induce cell injury, cells were treated with different concentrations of AA (0–80 μM) for the indicated time lengths over a 24-h time frame. Cells in the AA + Fer-1 group were pretreated with 5 µM Fer-1 for 6 h.

### 2.8 Cell viability assays

Cell viability was evaluated with Cell Counting Kit-8 (CCK-8) (Dojindo, Japan). Briefly, cells were inoculate in 96-well plates, and after exposure to different treatment conditions, 10 μL of CCK-8 solution was added to each well and incubated at 37°C in 5% CO2 for 1 h. The absorbance was measure at 450 nm using a SpectraMax microplate reader (Molecular Devices, Sunnyvale, CA).

### 2.9 Reactive oxygen species (ROS) detection

The intracellular ROS was assessed using a DCFDA (2′,7′-dichlorofluorescein diacetate) -Cellular ROS Assay Kit (Abcam, Cambridge, MA) according to the manufacturer’s instructions. Adherent cells were stained with 20 μM DCFDA for 45 min. After treatment, cells were detected in real time by fluorescence spectroscopy with excitation/emission at 485 nm/535 nm.

### 2.10 Iron detection

After treatment of cells with different substances, intracellular Fe^2+^ was labeled with 1 μmol/L FerroOrange (Dojindo, Japan) fluorescent probe. Then cells were incubated for 30 min and observed under a fluorescence microscope (Ex/Em = 546/580 nm).

### 2.11 Statistical analysis

At least three independent experiments were conducted for each experimental condition. Data are expressed as means ± SD. To compare between groups, the one-way analysis of variance (ANOVA) were performed. GraphPad Prism 5 software was used to process the data and to perform the statistical analyses.

## 3 Results

### 3.1 Inhibition of ferroptosis alleviated AA-induced AKI

**FIGURE 1:**
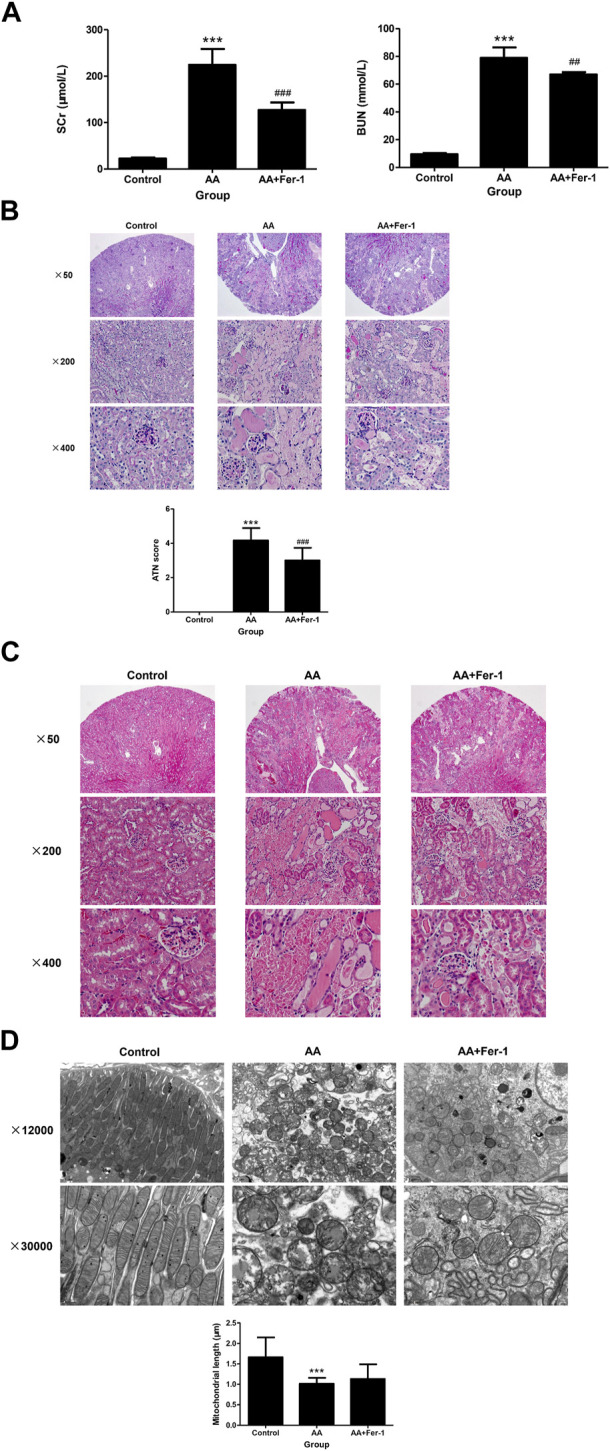
Renal function, renal histopathology, and renal electron microscopy of mice in each group. **(A)** SCr levels and BUN levels of the indicated groups. Data are reported as means ± SD (*n* = 5). ***p < 0.001 vs. the control group; ##*p* < 0.01, ###*p* < 0.001 vs. the AA group. Representative PAS staining **(B)** and HE staining **(C)** of the indicated groups. ATN score of the indicated groups. Data are reported as means ± SD (*n* = 5). ***p < 0.001 vs. the control group; ###*p* < 0.001 vs. the AA group. **(D)** Transmission electron microscopy of mitochondria and quantitative analysis of mitochondrial length among indicated groups. Data are reported as means ± SD (*n* = 43 in control group, *n* = 36 in AA group, and *n* = 29 in AA + Fer-1 group). ***p < 0.001 vs. the control group.

Compared with the control group, the serum creatinine (SCr) and blood urea nitrogen (BUN) levels were significantly higher in mice of AA group (224.3 ± 34.4 μmol/L vs. 22.6 ± 2.2 μmol/L, *p* < 0.001; 78.9 ± 7.6 mmol/L vs. 9.4 ± 0.87 mmol/L, *p* < 0.001), while mice in the AA + Fer-1 group had improved renal function, with significantly lower SCr and BUN levels compared with the AA group (127.0 ± 16.4 μmol/L vs. 224.3 ± 34.4 μmol/L, *p* < 0.001; 67.0 ± 1.7 mmol/L vs. 78.9 ± 7.6 mmol/L, *p* < 0.01) (Figure 1A). Histologically, the kidney structure of control group was normal, while the kidneys of AA group showed diffused acute tubular necrosis with coagulative necrosis and disintegration. The necrotic cell debris formed epithelial cell tubular pattern, and the tubules were compensated dilated. The necrotic lesions were especially obvious in the deep cortical layer and the corticomedullary junction (Figures 1B, C). The ATN score of the AA group was significantly higher than those of the control group (4.2 ± 0.72 vs. 0, *p* < 0.001). While in the AA + Fer-1 group, the ATN score was significantly decreased compared with the AA group (3.0 ± 0.74 vs. 4.2 ± 0.72, *p* < 0.001) (Figure 1B). Under the electron microscope, the mitochondria of renal tubular epithelial cells in mice of AA group showed necrotic morphology with sparse distribution. They were swollen and rounded, and those with severe necrosis were wrinkled and blurred. The mitochondrial cristae were reduced or even disappeared, and some of them showed outer membrane rupture. Quantitative analysis of mitochondrial morphology demonstrated that the average mitochondrial length decreased from 1.7 ± 0.5 to 1.0 ± 0.1 μm (*p* < 0.001). But treatment of Fer-1 did not show significant changes in mitochondrial length (1.1 ± 0.3 μm vs. 1.0 ± 0.1 μm) (Figure 1D). The overall lesions in the Fer-1 treated group were milder than those in the AA group. Focal tubular necrosis, detachment of the brush border and cellular vacuolation degeneration were seen with a smaller extent of lesions (Figures 1B, C).

### 3.2 Inhibition of ferroptosis alleviated AA-induced renal tubular epithelial cell injury

**FIGURE 2:**
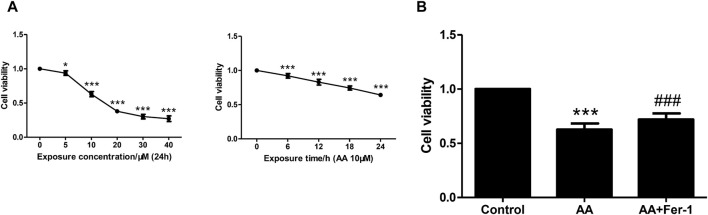
The effect of Fer-1 on cell viabilities in cells treated with AA. **(A)** Cells were treated with different concentrations of AA (0, 5, 10, 20, 30, 40 μM) for 24 h, or treated with 10 μM AA for the indicated times (0, 6, 12, 18, 24 h). Data are reported as means ± SD (*n* = 5). *p < 0.05, ***p < 0.001 vs. 0 h/0 μM. **(B)** Cells were treated with 10 μM AA for 24 h with or without Fer-1 pretreatment. Cell viabilities in each group were measured by CCK-8. ***p < 0.001 vs. the control group, ###*p* < 0.001 vs. the AA group (n = 5).

NRK-52E cells were incubated with various concentrations of AA (5, 10, 20, 30, 40 μM) for 24 h. The cell viability decreased with increasing concentration of AA. A significant decrease in cell viability was observed at a concentration of 5 μM (93.8% ± 3.3%, *p* < 0.05 vs. 0 μM). At a concentration of 10 μM, the cell viability decreased to 63.0% ± 3.7% (*p* < 0.001 vs. 0 μM) and to the lowest at a concentration of 40 μM (27.1% ± 4.1%, *p* < 0.001 vs. 0 μM) (Figure 2A). The cell viability also showed a time-dependent reduction with 10 μM AA treatment for 0–24 h (0, 6, 12, 18, 24 h). It decreased significantly from 6 h (92.2% ± 3.3%, *p* < 0.001 vs. 0 h), and to 64.2% ± 1.9% at 24 h (*p* < 0.001 vs. 0 h) (Figure 2A). Treatment of cells with 10 μM AA for 24 h resulted in a significant decrease in cell viability in the AA group (*p* < 0.001 vs. Control group), and the cell viability was significantly increased in the AA + Fer-1 group compared to the AA group from 62.7% ± 5.6% to 71.9% ± 5.7% (*p* < 0.001) (Figure 2B).

### 3.3 Inhibition of ferroptosis reduced lipid peroxidation in AA-induced AKI

**FIGURE 3:**
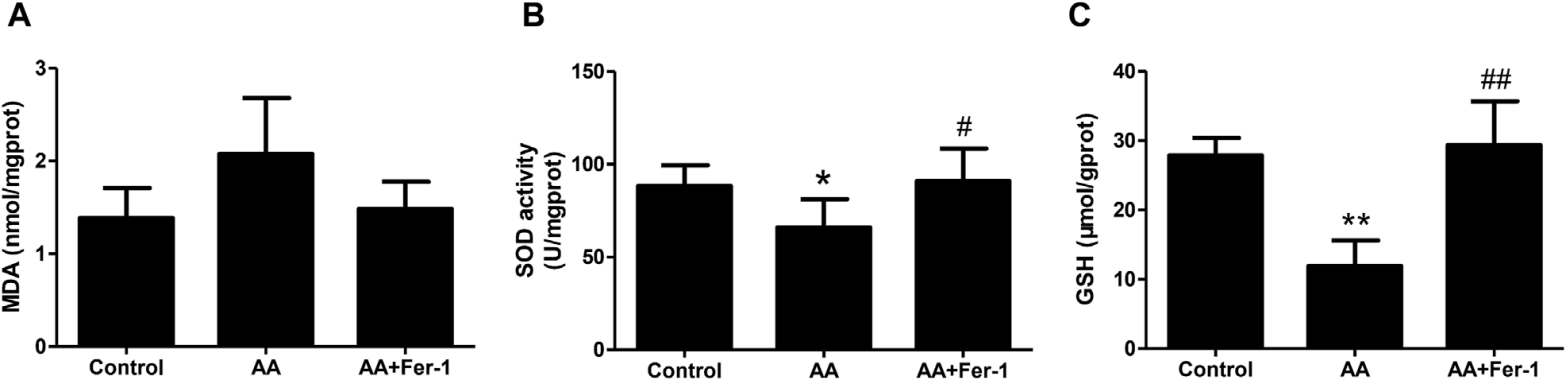
Inhibition of ferroptosis alleviates renal lipid peroxidation *in vivo*. **(A)** The MDA levels in kidney tissues. **(B)** The SOD activity in kidney tissues. **(C)** The GSH levels in kidney tissues. Data are reported as means ± SD (n = 5). *p < 0.05, **p < 0.01 vs. the control group; #*p* < 0.05, ##*p* < 0.01 vs. the AA group.

Compared with the control group, the MDA levels in kidney tissues was increased in the AA group (2.08 ± 0.60 nmol/mgprot vs. 1.39 ± 0.32 nmol/mgprot), and the SOD activity was reduced (65.9 ± 15.3 U/mgprot vs. 88.2 ± 11.3 U/mgprot). While the AA + Fer-1 group had lower MDA level (1.48 ± 0.29 nmol/mgprot vs. 2.08 ± 0.60 nmol/mgprot) and higher SOD activity (90.9 ± 17.6 U/mgprot vs. 65.9 ± 15.3 U/mgprot, *p* < 0.05) compared with AA group (Figures 3A, B). The GSH levels were significantly decreased in the AA group (11.9 ± 3.7 μmol/gprot vs. 27.9 ± 2.5 μmol/gprot, *p* < 0.01), while in the AA + Fer-1 group it was significantly increased compared with the AA group (29.4 ± 6.3 μmol/gprot vs. 11.9 ± 3.7 μmol/gprot, *p* < 0.01) (Figure 3C). These results suggest that Fer-1 improved the lipid peroxidation status in AAN mice.

**FIGURE 4:**
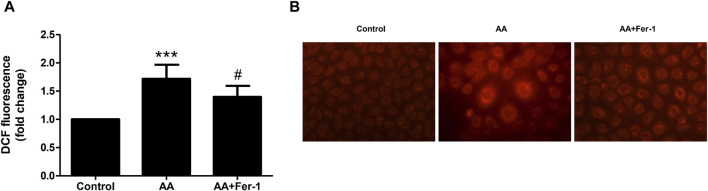
Inhibition of ferroptosis reduced ROS production and iron content *in vitro*. **(A)** Cells were treated with 10 μM AA for 1 h with or without Fer-1 pretreatment. The intracellular ROS was assessed using DCFDA cellular ROS assay. Data are reported as means ± SD (*n* = 5). ***p < 0.001 vs. the control group, #*p* < 0.05 vs. the AA group. **(B)** Cells were treated with 10 μM AA for 24 h with or without Fer-1 pretreatment. The intracellular Fe^2+^ was detected using FerroOrange fluorescent probe (×400).

When NRK-52E cells were treated with 10 μM AA for 1 h, the ROS levels significantly elevated (*p* < 0.001 vs. Control group). And by applying Fer-1, the ROS levels were markedly decreased compared to the AA group (1.4 ± 0.2-fold vs. 1.7 ± 0.3-fold, *p* < 0.05) (Figure 4A). In addition, the intracellular Fe^2+^ fluorescence intensity was increased by 10 μM AA incubation for 24 h. After Fer-1 treatment, a decrease in cell fluorescence intensity was observed, and the proportion of cells with stronger fluorescence signal was less seen (Figure 4B).

### 3.4 Inhibition of ferroptosis restored GPX4 protein expression and decreased ACSL4 protein expression in AA-induced AKI

**FIGURE 5:**
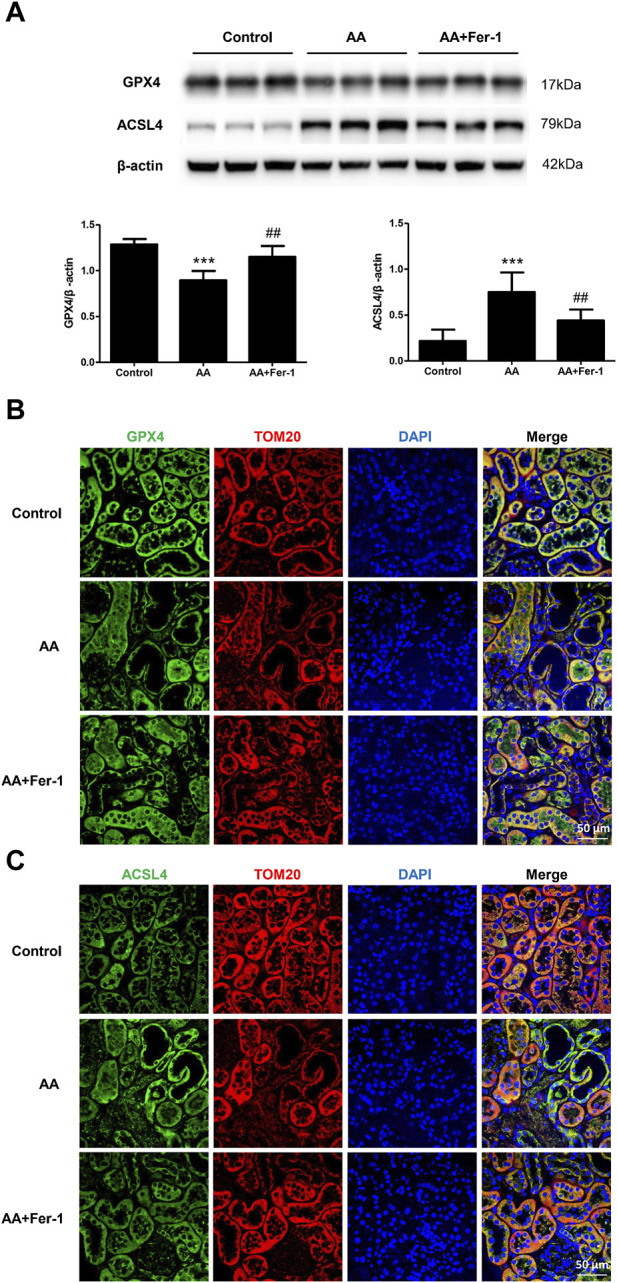
The effect of Fer-1 on GPX4 and ACSL4 protein expression in kidney tissues. **(A)** Western blotting analysis of GPX4 and ACSL4 in each group was shown. Densitometric analysis of protein expression was normalized to β-actin content. Data are reported as means ± SD (*n* = 5). ***p < 0.001 vs. the control group, ##*p* < 0.01 vs. the AA group. **(B)** Representative immunofluorescence staining of GPX4 (green) in kidney tissues of each group (×400). **(C)** Representative immunofluorescence staining of ACSL4 (green) in kidney tissues of each group (×400).

In the AA group, the GPX4 protein expression in the kidney tissue was significantly lower (*p* < 0.001), which mainly distributed in the cytoplasm of renal tubular epithelial cells. It mainly expressed in the residual tubular cells, and the fluorescence signal was diminished. Compared with the AA group, the GPX4 protein expression was significantly increased in the AA + Fer-1 group (*p* < 0.01), and the immunofluorescence signal was enhanced (Figures 5A, B). The ACSL4 protein expression was significantly increased in the kidney tissue of the AA group (*p* < 0.001), which mainly distributed in the renal tubular epithelial cell membrane and cytoplasm, and the fluorescence signal was enhanced in the injured renal tubules. While the ACSL4 protein expression was significantly decreased in the AA + Fer-1 group (*p* < 0.01) compared with the AA group, and the immunofluorescence signal was diminished (Figures 5A, C).

**FIGURE 6:**
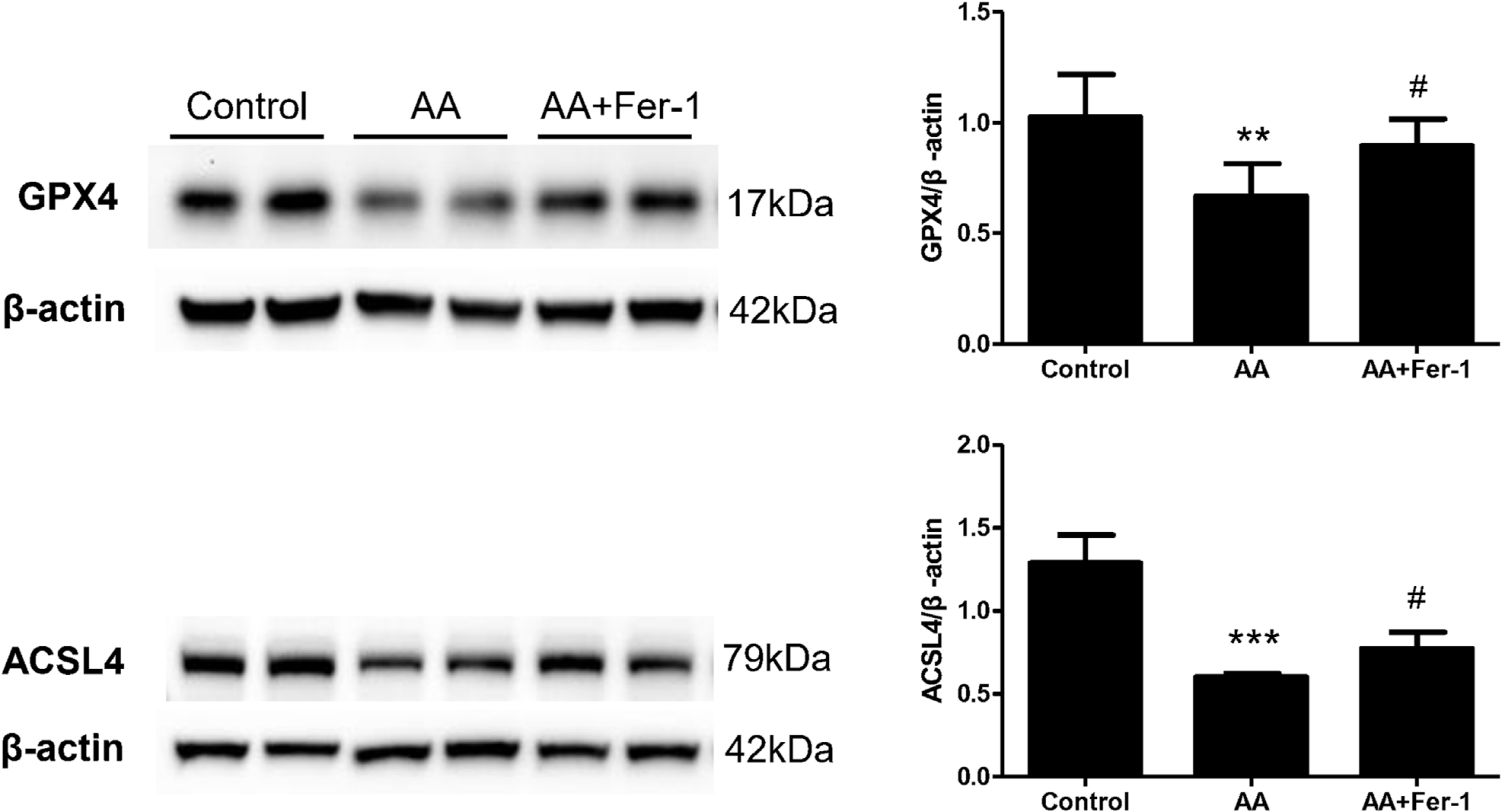
The effect of Fer-1 on GPX4 and ACSL4 protein expression in cells treated with AA. Cells were treated with 10 μM AA for 24 h with or without Fer-1 pretreatment. Western blotting analysis of GPX4 and ACSL4 in each group was shown. Densitometric analysis of protein expression was normalized to β-actin content. Data are reported as means ± SD (*n* = 3). **p < 0.01, ***p < 0.001 vs. the control group; #*p* < 0.05 vs. the AA group.

In NRK-52E cells, the expression of ACSL4 proteins showed a time-dependent reduction when cells were treat with 10 μM AA for 0–24 h (0, 6, 12, 18, 24 h) (Supplementary Figure S1). Treatment of cells with 10 μM AA for 24 h caused a significant decrease in the expression of GPX4 and ACSL4 (*p* < 0.01 vs. Control group; *p* < 0.001 vs. Control group), while Fer-1 treatment increased the expression of GPX4 and ACSL4 proteins significantly (*p* < 0.05, vs. AA group; *p* < 0.05 vs. AA group) (Figure 6).

## 4 Discussion

In the present study, we found that the acute AAN mice showed increased MDA levels, aggravated lipid peroxidation, decreased SOD activity, GSH depletion, and impairment of antioxidant capacity in the kidney tissues. The expression of endogenous ferroptotic inhibitor GPX4 was decreased and the expression of phospholipid synthesis-related enzyme ACSL4 was increased. It is suggested that ferroptosis is involved in AA-induced AKI. Inhibition of ferroptosis by Fer-1 significantly improved the renal function, reduced histopathological lesions, partially alleviated lipid peroxidation, and restored the antioxidant capacity. *In vitro* studies also showed that AA significantly reduced cell viability, induced ROS production, increased intracellular iron level and decreased ferroptosis-related protein expression. Inhibition of ferroptosis significantly increased cell viability and attenuated AA-induced renal tubular epithelial cell injury.

As the main endogenous antioxidant enzyme, GPX4 prevents the accumulation of toxic lipid ROS during ferroptosis. Depletion of GSH and inactivation of GPX4 will result in iron-dependent accumulation of lipid ROS, which is lethal to cells (Dixon et al., 2012). Direct inhibition of GPX4 can lead to ferroptosis. Gpx4−/− mice exhibit complete penetrant embryonic lethality (Imai et al., 2003; Yant et al., 2003), and conditional knockout of Gpx4 is associated with cancer, neurodegenerative diseases, acute kidney injury or liver injury, which can be prevented or mitigated by inhibiting ferroptosis (Imai et al., 2003; Seiler et al., 2008; Carlson et al., 2016; Doll et al., 2017; Hambright et al., 2017). Our *in vivo* and *in vitro* studies also revealed a significant reduction in GPX4 expression in the AA-induced acute tubular injury model. It mainly expressed in the residual tubular epithelial cells, accompanied by a significant decrease in GSH levels, suggesting an impairment of the antioxidant function. Inhibition of ferroptosis resulted in a significant increase in GPX4 expression and GSH levels. It is suggested that AA may affect the metabolism of GSH and induce ferroptosis by directly or indirectly inhibiting GPX4, while inhibition of ferroptosis reduces renal damage by restoring the antioxidant capacity.

The key feature of ferroptosis is the iron-dependent lipid peroxidation process. The presence of sufficient free intracellular iron and polyunsaturated fatty acids (PUFA) in the cell membrane are both prerequisites for ferroptosis. Iron chelators or lipophilic antioxidants can effectively prevent lipid ROS accumulation and cell death. Both acyl-CoA synthetase long-chain family member 4 (ACSL4) and lysophosphatidylcholine acyltransferase 3 (LPCAT3) are involved in the synthesis and remodeling of PUFA-phosphatidylethanolamines (PEs) in cellular membranes. Genetic or pharmacological inhibition of ACSL4, rather than other members of the ACSL family, prevents cells from undergoing ferroptosis. Therefore, ACSL4 is an essential component in the process of ferroptosis (Doll et al., 2017). In addition, ferroptosis is usually associated with a disturbance of iron homeostasis, which leads to an abnormal increase in free cellular iron concentration. Lipoxygenase or excessive free iron oxidizes PUFAs on the membrane through enzymatic or nonenzymatic reactions, leading to lipid ROS formation which promotes ferroptosis.

In this study, we found that the expression of ACSL4 protein was significantly increased in the kidney tissue of AAN mice, and the fluorescence signal of the injured tubules was enhanced. Meanwhile, the MDA level of kidney tissue was increased and the SOD activity was significantly decreased. *In vitro* studies also showed that the cellular ROS production was significantly increased after AA treatment, accompanied by an increase in intracellular Fe^2+^ level. It is suggested that more membrane phospholipids which are susceptible to oxidation are synthesized. Thus the level of lipid peroxidation is increased, leading to an increase in its metabolites. While inhibition of ferroptosis resulted in a significant decrease in ACSL4 expression, decreased MDA levels, increased SOD activity, and decreased cellular ROS production, which partially reduced the level of lipid peroxidation. It is indicated that AA may trigger ferroptosis by inducing iron-dependent lipid peroxidation. And Fer-1 alleviated kidney injury by scavenging excess lipid peroxides. But the specific mechanism of abnormal increase in free iron induced by AA needs to be further investigated. In contrast to the *in vivo* study, our *in vitro* study showed that ACSL4 protein levels were decreased after AA treatment and Fer-1 treatment increased ACSL4 protein expression. Other studies have also shown similar results, where ACSL4 protein expression was elevated in renal ischemia reperfusion injury (IRI) mice models, and ferroptosis inhibitor 16–86 decreased ACSL4 protein levels. *In vitro*, erastin induced a time-dependent reduction in ACSL4 protein expression in NIH3T3 cells, and Fer-1 prevented the ACSL4 protein from decreasing. Due to the continuous depletion of membrane phospholipids in response to ongoing lipid peroxidation, ACSL4 protein is compensatorily expressed *in vivo* in order to repair plasma membrane damage, suggesting that ACSL4 expression is upregulated during nonlethal ferroptosis. Whereas ACSL4 protein in *in vitro* cells is constantly depleted with lipid peroxidation, hence ACSL4 expression is downregulated after ferroptosis initiates cell death (Mueller et al., 2017).

Ferroptosis was identified in various forms of AKI. GPX4 knockdown led to AKI and lipid peroxidation in mice, and ferroptosis inhibitor Fer-1, attenuated kidney injury and protected renal function (Friedmann et al., 2014), suggesting that ferroptosis may be a potential therapeutic target for AKI. In severe IRI mice models, application of Fer-1 ameliorated renal function and kidney injury, whereas the receptor-interacting protein kinase (RIPK) 1 inhibitor Nec-1 failed to protect against hypoxic injury of renal tubules. This suggests that inhibition of ferroptosis is more protective than inhibition of necroptosis in renal IRI (Linkermann et al., 2014). In folic acid-induced AKI, ferroptosis is the main regulated necrosis pathway. Inhibition of ferroptosis with Fer-1 improved renal function and reduced tissue damage. However, inhibition of necroptosis or apoptosis at pharmacological or genetic level was not found protective (Martin-Sanchez et al., 2017). Baliga et al. reported that exposure to cisplatin led to a significant increase in iron levels in *in vitro* and *in vivo* AKI models. The use of deferoxamine significantly improved renal function and alleviated tissue damage (Baliga et al., 1998). Fer-1 pretreatment attenuated cisplatin-induced renal injury in mice and improved renal function. *In vitro*, Fer-1 reduced cisplatin-induced HK-2 cell death, while Nec-1 had no protective effect (Deng et al., 2019). In rhabdomyolysis (RM)-induced AKI, only Fer-1 attenuated oxidative stress, reduced cell death, and protected renal function, while caspase inhibitors zVAD or RIPK3 knockdown did not show protect effect (Homsi et al., 2015). Regarding the role of ferroptosis in acute AAN, an *in vitro* study showed that aristolactam I (ALⅠ), a metabolite of AA, significantly inhibited GSH levels in HK-2 cells, accompanied by an increase in intracellular 4-HNE and ferric ions, mitochondrial iron overload, and decreased GPX4 protein expression. Iron chelator DFO significantly attenuated cytotoxicity. Furthermore, the use of Fer-1 improved cell viability and increased GPX4 protein expression to a greater extent compared to the apoptosis inhibitor zVAD or necrosis inhibitor Nec-1. It is implied that ferroptosis is the main cell death pathway of ALⅠ-induced cell death (Deng et al., 2020). In addition, metabolomic analysis revealed that AAN rats exhibited activation of oxidative stress and inflammatory pathways, and these changes were accompanied by downregulation of glutamate-cysteine ligase, SOD and catalase. The metabolism of fatty acids, phospholipids and glycerolipids in renal tissues was remarkably changed, with a significant increase in fatty acid concentrations that may be associated with oxidative fatty acid damage (Zhao et al., 2015).

In conclusion, this study indicated that ferroptosis plays an important role in AA-induced acute tubular injury. And inhibition of ferroptosis may exert renoprotective effects possibly by preventing lipid peroxidation, restoring the antioxidant activity or regulating iron metabolism. Regulation of necrotic cell death signaling such as ferroptosis may be an effective way in intervention of AKI.

## Data Availability

The original contributions presented in the study are included in the article/Supplementary Material, further inquiries can be directed to the corresponding author.

## Supplementary material

supplementary figure S1 The expression of ACSL4 protein in cells treated with AA. Cells were treated with 10 μM AA for 0–24 h (0, 6, 12, 18, 24 h). Western blotting analysis of ACSL4 in each group was shown. Densitometric analysis of protein expression was normalized to β‐actin content. Data are reported as means ± SD (n = 3). *p < 0.05, **p < 0.01, ***p < 0.001 vs. 0 h.
