# Supplementary figures and images for: Aristolochic acid induces acute kidney injury through ferroptosis
Source: Front Pharmacol. 2024 Mar 27;15:1330376. doi: 10.3389/fphar.2024.1330376 (PMC11004286; doi:10.3389/fphar.2024.1330376)

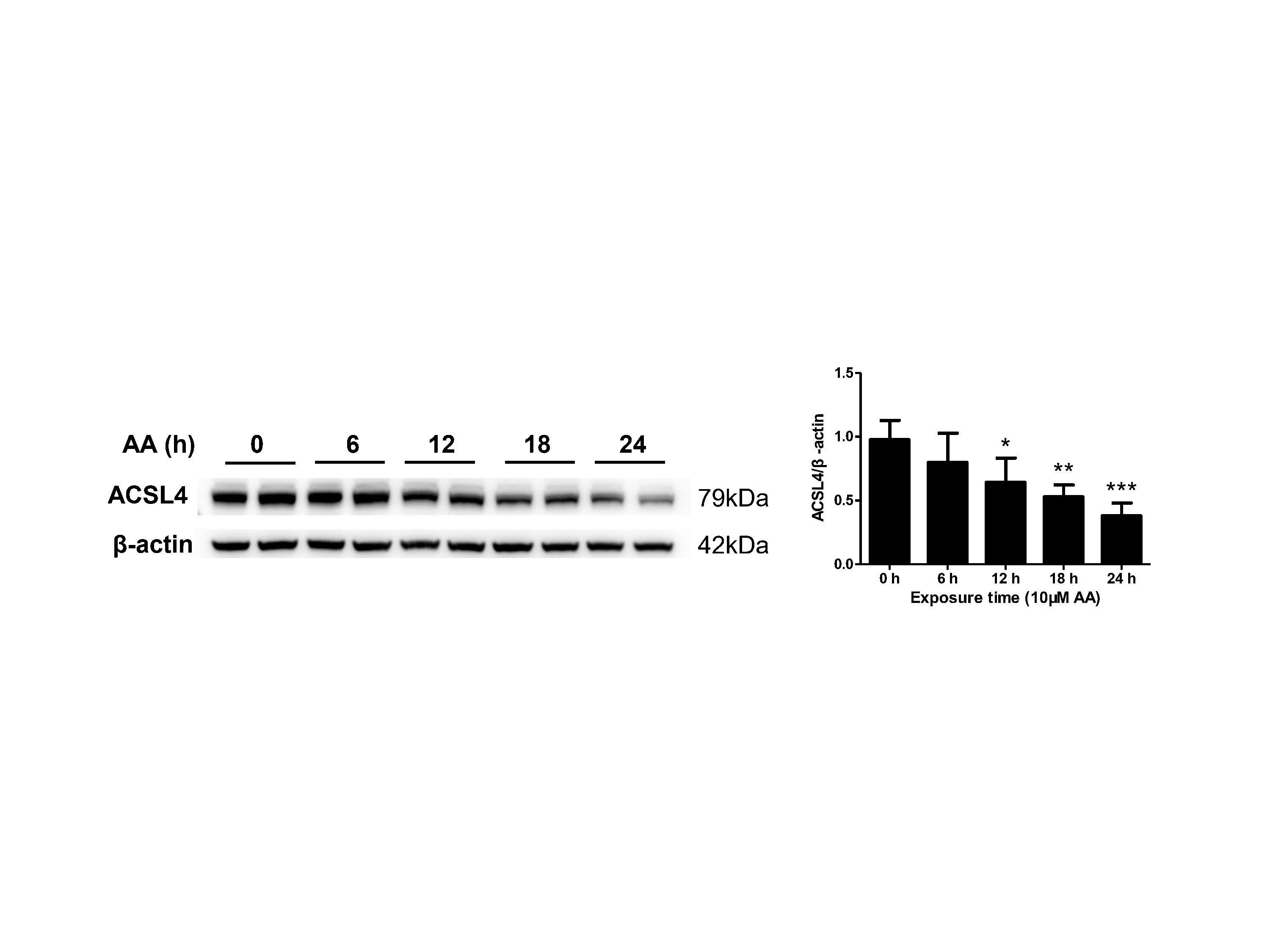

Supplement: Supplementary file 1 [file Image1.TIF]
